# Supplementary material for: Self-organization of primitive metabolic cycles due to non-reciprocal interactions
Source: Nat Commun. 2023 Jul 26;14:4496. doi: 10.1038/s41467-023-40241-w (PMC10372013; doi:10.1038/s41467-023-40241-w)
Supplement: Supplementary file 1 — Supplementary information [file 41467_2023_40241_MOESM1_ESM.pdf]

Supplementary Information
Self-organization of primitive metabolic cycles due to non-reciprocal interactions

Vincent Ouazan-Reboul,[1] Jaime Agudo-Canalejo,[1] and Ramin Golestanian[1,2]

[1] *Max Planck Institute for Dynamics and Self-Organization, Am Fassberg 17, D-37077, Göttingen, Germany*

[2] *Rudolf Peierls Centre for Theoretical Physics, University of Oxford, OX1 3PU, Oxford, UK*

CONTENTS

Supplementary Note 1: Eigenvalue spectra 2

Supplementary Note 2: Oscillatory dynamics for an odd number of chasing species 2

Additional simulation snapshots 3

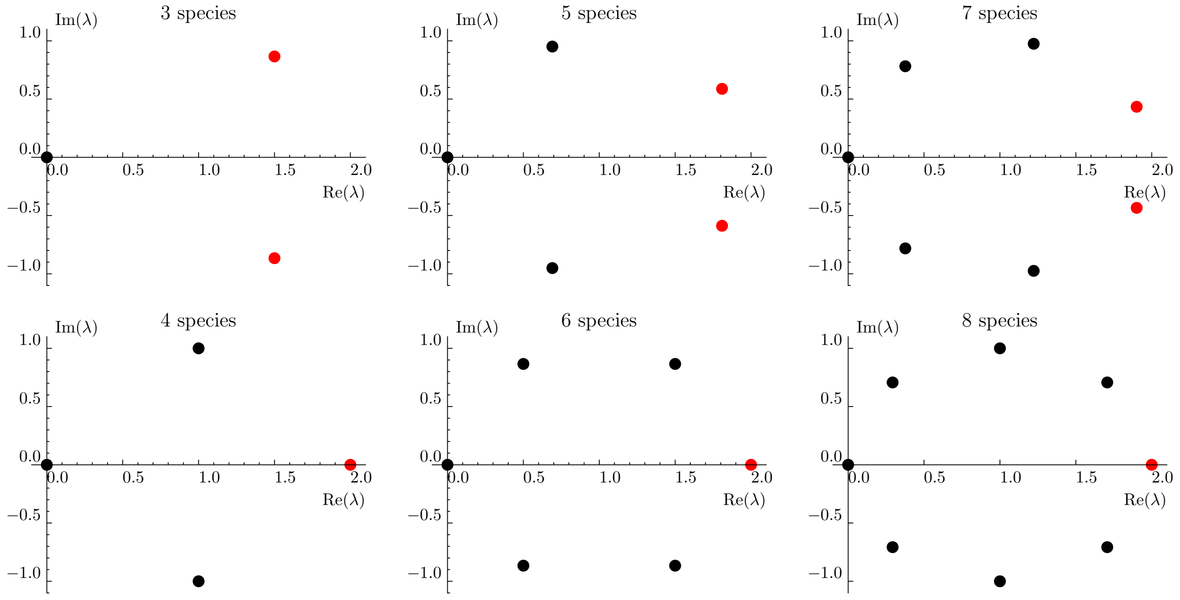

Supplementary Figure 1. Eigenvalue spectra for $M = 3$ to 8 species, in units of $-(\mu^{(p)} - \mu^{(s)})\alpha\rho_0/D$ for the real part and $-(\mu^{(s)} + \mu^{(p)})\alpha\rho_0/D$ for the imaginary part. The eigenvalue (for an even number of species) or pair of eigenvalues (for an odd number) with the largest real part is coloured in red.

SUPPLEMENTARY NOTE 1: EIGENVALUE SPECTRA

In Supplementary Figure 1, we plot in the complex plane the spectrum of the stability matrix (equation (3) of the main text) for different numbers of species M, which corresponds to the M eigenvalues given in equation (4) of the main text. These spectra illustrate the key difference between even and odd species numbers, as highlighted in the main text: for cycles involving an even number of species, the fastest growing mode is associated to a real eigenvalue, while for cycles involving an odd number of species, the fastest growing mode is associated to a complex conjugate pair of eigenvalues. In the latter case, the ratio between the magnitudes of the real and imaginary parts (see the full expression given in equation (8) of the main text) determines whether the system exhibits long-lived oscillations.

SUPPLEMENTARY NOTE 2: OSCILLATORY DYNAMICS FOR AN ODD NUMBER OF CHASING SPECIES

Using the clustering scheme described in Methods, we quantify the oscillation dynamics shown in Fig. 4 of the main text and Supplementary Movie 8. For each species, we plot in Supplementary Figure 2 the normalized mean size of the clusters containing at least one particle of that species, excluding individual particles (i.e. clusters of size 1). A uniform moving average is applied over a 10-point window in order to smooth out high-frequency variations.

In accordance with Fig. 4 of the main text and Supplementary Movie 8, we observe successive formation and dissolution of large clusters containing a given species, in the order $\cdots \to 5 \to 4 \to 3 \to 2 \to 1 \to 5 \to \ldots$. These formation and explosion events are regularly spaced in time, and can be interpreted as constituting an oscillatory steady state. At their largest, the clusters span 20 to 30 % of the total particle population, reflecting the fact that two clusters of species of the same parity might be in contact right before an explosion event, as shown in Fig. 4 of the main text.

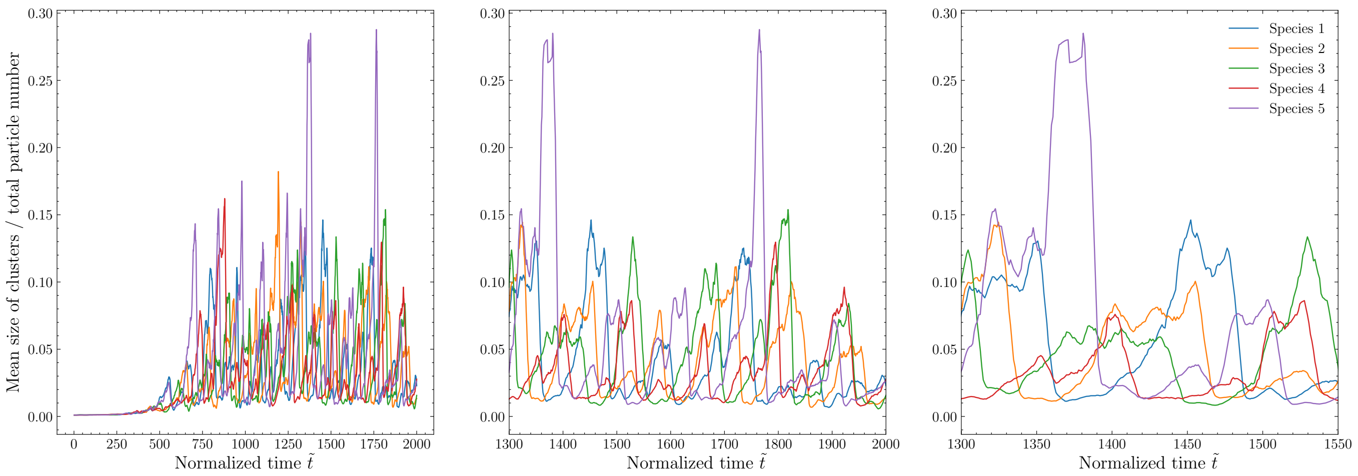

Supplementary Figure 2. Oscillation dynamics for $M = 5$ species (see Fig. 4 of the main text and Supplementary Movie 8). Left: full time span of the simulation. Centre: five oscillation cycles. Right: two oscillation cycles.

ADDITIONAL SIMULATION SNAPSHOTS

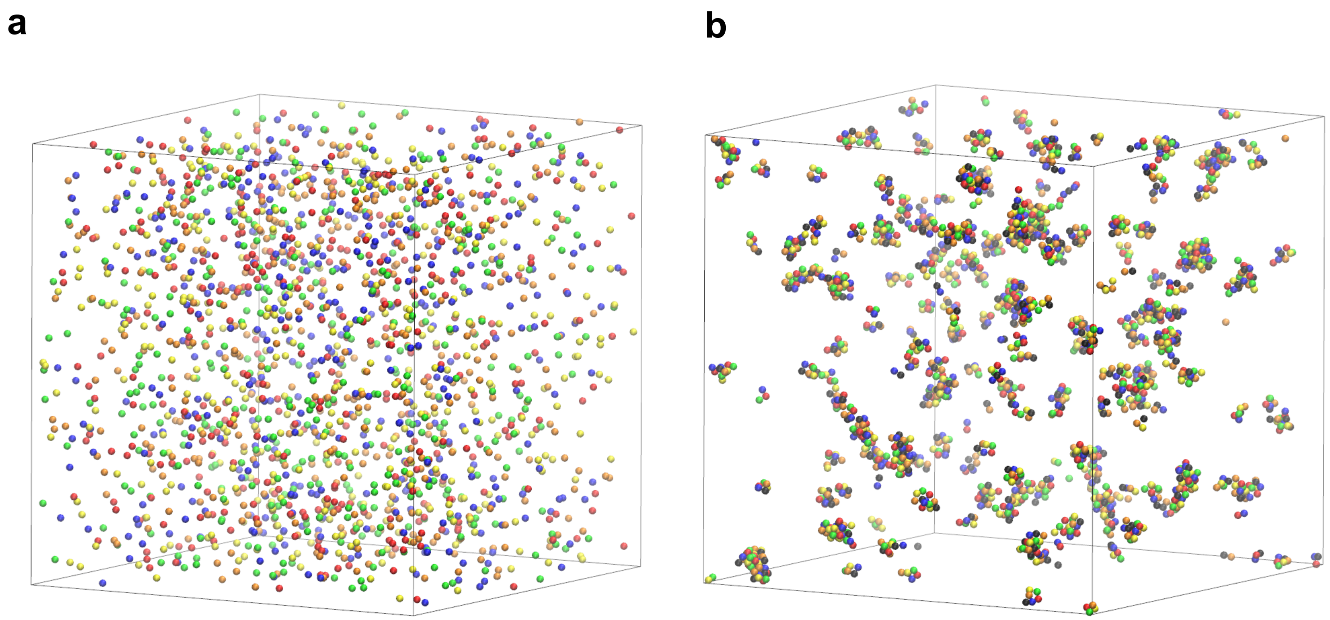

Supplementary Figure 3. Different types of homogeneous steady states. **a**: Self-repelling, cross-chasing regime (Supplementary Movie 1). Particles form short-lived, self-propelled molecules, which swap particles with each other. **b**: Self-repelling, cross-attracting regime (Supplementary Movie 2). Particles form larger, long-lived, rotating molecules. If $\mu^{(s)} = -\mu^{(p)}$, rotation is suppressed.

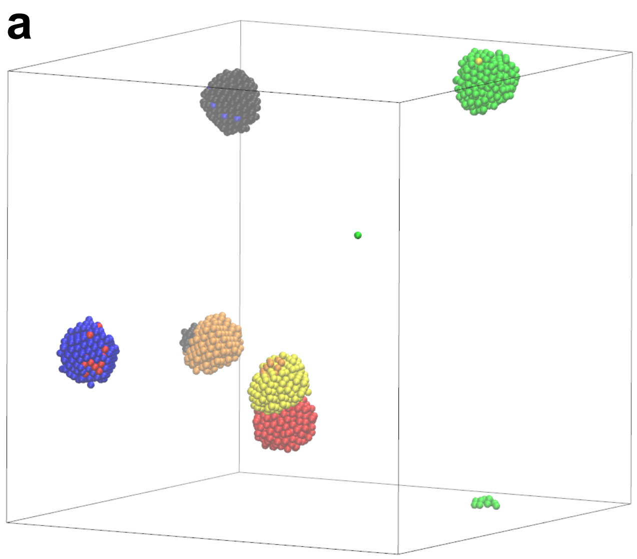

Supplementary Figure 4. Chasing regime for $M = 6$ particle species. **a**: Weak chasing, strong self-attraction regime (Supplementary Movie 4). Species aggregate in mixed clusters without oscillations. Each cluster is composed of a majority species and a minority species that chases the majority one. Because of their mixed composition, the clusters chase each other over long timescales. Transient aggregation of the clusters is observed. **b**: Strong chasing, weak self-attraction regime (Supplementary Movie 6). After transient oscillations, two clusters form, one containing a mixture of all even species, the other of all odd species.

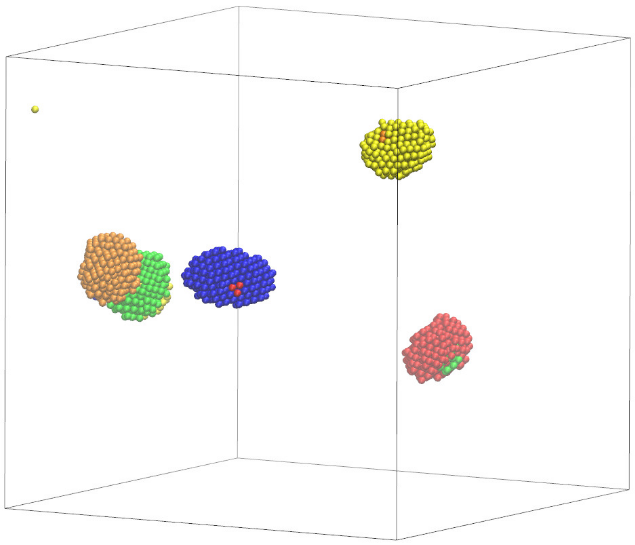

Supplementary Figure 5. Non-oscillatory steady state for $M = 5$ particle species. Species aggregate into mixed clusters, which then chase each other (Supplementary Movie 10).
