# Supplementary material for: Self-organization of primitive metabolic cycles due to non-reciprocal interactions
Source: Nat Commun. 2023 Jul 26;14:4496. doi: 10.1038/s41467-023-40241-w (PMC10372013; doi:10.1038/s41467-023-40241-w)
Supplement: Supplementary file 3 — Description of Additional Supplementary files [file 41467_2023_40241_MOESM3_ESM.pdf]

Description of Additional Supplementary Files
Self-organization of primitive metabolic cycles due to non-reciprocal interactions

Vincent Ouazan-Reboul,[1] Jaime Agudo-Canalejo,[1] and Ramin Golestanian[1,2]

[1] *Max Planck Institute for Dynamics and Self-Organization, Am Fassberg 17, D-37077, Göttingen, Germany*

[2] *Rudolf Peierls Centre for Theoretical Physics, University of Oxford, OX1 3PU, Oxford, UK*

- **File Name: Supplementary Movie 1**
  Description: Linearly stable mixture of self-repelling species in the chasing regime, resulting in the formation of transient self-propelled colloidal molecules. See Supplementary Fig. 3a.
- **File Name: Supplementary Movie 2**
  Description: Linearly stable mixture of self-repelling, cross-attracting particle species, which form long-lived rotating molecules. See Supplementary Fig. 3b.
- **File Name: Supplementary Movie 3**
  Description: Unstable mixture of an even number of self-attracting, cross-repelling particle species undergoing parity-based aggregation. See Fig. 3a of the main text.
- **File Name: Supplementary Movie 4**
  Description: Linearly unstable mixture of an even number of self-attracting, cross-chasing species for which the magnitude of the chasing interaction is comparable to the self-attraction. The mixture self-organizes into hybrid clusters composed of a majority and a minority species which chase each other. See Supplementary Fig. 4a.
- **File Name: Supplementary Movie 5**
  Description: Same as Supplementary Movie 4, but with the magnitude of the chasing interaction being larger than the self-attraction. Two hybrid clusters, each containing all the species of a same parity, form and separate.
- **File Name: Supplementary Movie 6**
  Description: Same as Supplementary Movie 4, but with self-attraction negligible compared to chasing interactions. The system behaves similarly to Supplementary Movie 5, with transient oscillations observed as the clusters form. See Supplementary Fig. 4b.
- **File Name: Supplementary Movie 7**
  Description: Linearly unstable mixture of an odd number of self-attracting, cross-repelling species. The particles separate into single-species clusters, in order to minimize overall repulsion. See Fig. 3b of the main text.
- **File Name: Supplementary Movie 8**
  Description: Linearly unstable mixture of an odd number of self-attracting, cross-chasing species, with a magnitude of the self-attraction smaller, but on the same order as the chasing interaction. The system exhibits long-lived oscillations in which species successively form clusters which then get dissolved and replaced by a chasing species. See Fig. 4 of the main text.
- **File Name: Supplementary Movie 9**
  Description: Same as Supplementary Movie 8, but with the self-attraction magnitude taken much lower than the chasing interaction. The system behaves similarly to Supplementary Movie 8, with the clusters being replaced by regions of transiently increased concentration.
- **File Name: Supplementary Movie 10**
  Description: Same as Supplementary Movie 8, but in a parameter regime for which the instability growth rate is larger than its oscillation frequency. The particles phase-separate into hybrid clusters of two species without exhibiting oscillations. See Supplementary Fig. 5.
